# Ecology dictates the value of memory for foraging bees

**DOI:** 10.1016/j.cub.2022.07.062

**Published:** 2022-10-10

**Authors:** Christopher D. Pull, Irina Petkova, Cecylia Watrobska, Grégoire Pasquier, Marta Perez Fernandez, Ellouise Leadbeater

**Affiliations:** 1 Department of Biological Sciences, Royal Holloway University of London, Egham, Surrey TW20 0EX, UK; 2 Department of Geography, Royal Holloway University of London, Egham, Surrey TW20 0EX, UK

**Keywords:** ecological intelligence, short-term memory, evolutionary cognition, radial arm maze, bumblebee foraging, floral phenology

## Abstract

“Ecological intelligence” hypotheses posit that animal learning and memory evolve to meet the demands posed by foraging and, together with social intelligence and cognitive buffer hypotheses, provide a key framework for understanding cognitive evolution.^1^, ^2^, ^3^, ^4^, ^5^ However, identifying the critical environments where cognitive investment reaps significant benefits has proved challenging.^6^, ^7^, ^8^ Here, we capitalize upon seasonal variation in forage availability for a social insect model (*Bombus terrestris audax*) to establish how the benefits of short-term memory, assayed using a radial arm maze (RAM), vary with resource availability. Following a staggered design over 2 years, whereby bees from standardized colonies at identical life-history stages underwent cognitive testing before foraging in the wild, we found that RAM performance predicts foraging efficiency—a key determinant of colony fitness—in plentiful spring foraging conditions but that this relationship is reversed during the summer floral dearth. Our results suggest that the selection for enhanced cognitive abilities is unlikely to be limited to harsh environments where food is hard to find or extract,^5^^,^^9^, ^10^ highlighting instead that the challenges of rich and plentiful environments, which present multiple options in short succession, could be a broad driver in the evolution of certain cognitive traits.

**Video abstract:**

## Highlights

• Bees learn about floral rewards when foraging
• Bees with better memory scores foraged more efficiently in spring but not in summer
• Spring floral environments are rich and complex, but summer ones are sparse
• Plentiful environments with many foraging options may favor cognitive evolution

## Graphical abstract

**Figure:**
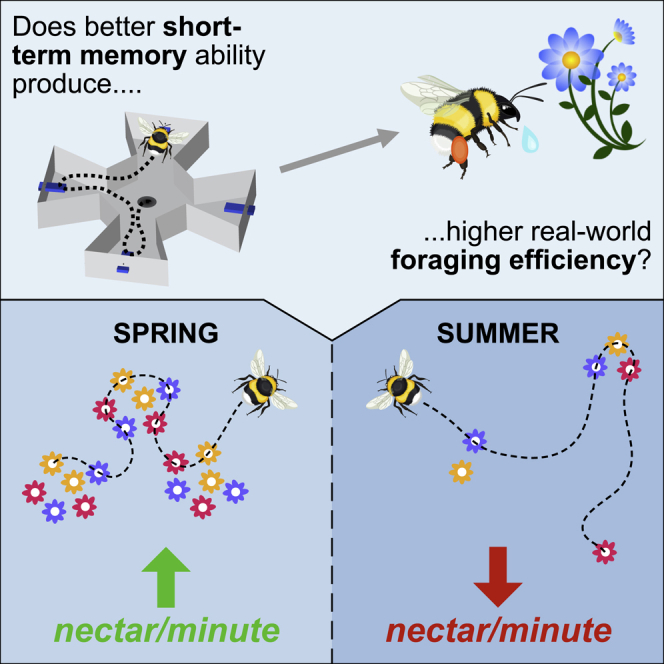

## Teaser

Using a maze designed to assay shorter-term memory in bees that later forage in the wild, Pull et al. find that high-performance bees are more efficient foragers in spring, when floral resources are rich, but not in summer, when resources are scarcer, challenging the notion that harsh environments primarily select for cognitive traits.

## Results

The spatiotemporal distribution of food has been repeatedly theorized to contribute to the evolution of cognitive traits.^1^, ^2^, ^3^, ^4^, ^5^ In particular, the gross benefits of investment into learning and memory have been proposed to outweigh the significant constitutive and induced costs that these traits carry^11^^,^^12^ when food is hard to find because it is scarce, heterogeneous, novel, or challenging to extract.^4^^,^^10^^,^^13^^,^^14^ Accordingly, comparative neuroanatomical studies have linked potentially demanding foraging tasks, such as remembering the location of cached food during high-elevation winters, to changes in size or structure of neural regions across species.^13^^,^^15^, ^16^, ^17^ However, the considerable challenges of standardizing confounding noncognitive factors (e.g., previous experience, parasite load, or motivation) that can influence cognitive assay performance^6^, ^7^, ^8^ when working with wild animals, mean that direct evidence to link cognitive abilities to fitness proxies is still rare. Those studies that have overcome such hurdles^10^^,^^18^^,^^19^ have not been extended to include ecological variation across environments, which is predicted to be a fundamental driver of interspecific variation in cognitive traits.^7^^,^^8^^,^^20^^,^^21^ As such, we do not yet have a full picture of when cognitive abilities are most valuable in the wild and thus of the ecological conditions that favor cognitive evolution.

Here, we capitalize upon temporal variation in food availability within the colony lifespan of a social insect model to examine how the benefits afforded by a specific cognitive trait may vary with resource availability. *Bombus* are a temperate group and in most species workers begin to emerge in the early spring.^22^ Since the maximum lifespan of individual workers in the field is typically no more than 4 weeks,^23^, ^24^, ^25^ foraging is carried out by successive overlapping worker generations in most species, until the production of reproductive offspring and subsequent colony death in late summer. The availability of floral forage varies considerably across this time, typically reaching a peak in the spring that recedes to a trough in late summer, with local variations.^26^ Foraging bees can visit hundreds of individual flowers across repeated foraging bouts every day, and cognitive traits such as learning and memory are thought likely to be fundamental in maximizing colony foraging success through their impacts on foraging efficiency.^27^^,^^28^ However, despite a rich history of investigation into the mechanisms and role of memory for foraging bees,^29^, ^30^, ^31^ few studies have empirically tested hypothesized links between memory performance and real-world foraging efficiency and those that have done so have focused on short summer foraging windows^19^^,^^32^ and thus do not capture any ecological variation in resource availability.

The neurological basis of memory formation is well described in bees,^29^^,^^33^ and different phases of memory formation may be relevant to different aspects of foraging that reflect the patchy distribution of floral resources.^28^ For example, medium- and long-term memories that last in the region of hours to days may allow bees to remember the locations of rewarding flower patches, and the identities of nectar- or pollen-rich flower types because they both depend on transcription and translation, which stabilize memories in time.^28^^,^^31^^,^^34^ In contrast, short-term memories that last seconds to minutes and require neither transcription nor translation to form^29^ may be more relevant to within-patch foraging,^28^ where flight times between flowers are typically a few seconds.^35^ In this context, memories of the most recently visited flowers might allow bees to temporarily avoid previously visited and depleted flowers as they quickly move between them (as demonstrated under experimental conditions^22^^,^^36^, ^37^, ^38^) and to decide whether to stay in a patch and remain constant to a flower species.^28^^,^^35^^,^^39^^,^^40^ Here, we focus on the relationship between bumblebee foraging efficiency and memory performance in the shorter within-patch time frame, across a highly variable foraging season.

**Figure 1:**
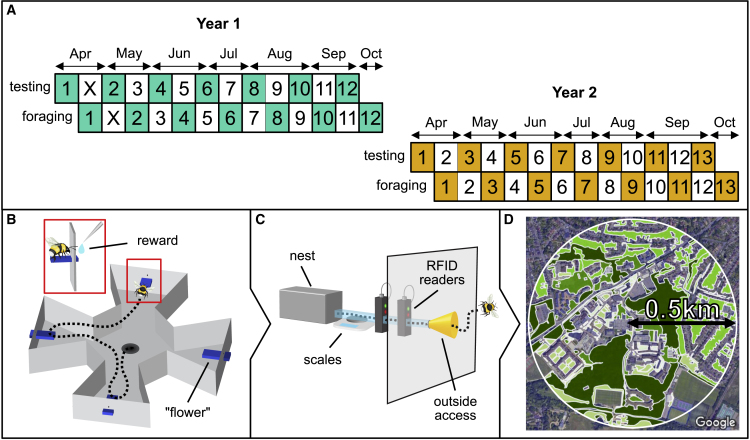
Lab-to-field testing of bee cognition and foraging efficiency (A) Experimental timeline showing staggered design: each number indicates a colony (n = 25; colony X died during testing). (B) Four-arm radial arm maze (RAM) used to assay short-term memory (STM); inset shows bee extracting a reward. (C) Bees were weighed on exit and return to the nest, passing through RFID readers. (D) Colonies were surrounded by university parkland, suburban greenspace, and private gardens. The most dominant land type was mixed woodland (dark green). Non-colored areas represent areas unlikely to contain floral resources. See also Figure S1 for RAM validation and performance and Data S1A for model selection outcomes. Satellite imagery from Google Maps (Imagery ©2022 Getmapping plc, Infoterra Ltd & Bluesky, Maxar Technologies, The GeoInformation Group, Map data ©2022).

Over two successive years, we reared 25 young, commercially supplied colonies in succession under identical aseasonal laboratory conditions from April to September. Within each year, we followed a staggered design (Figure 1A) such that each colony began the testing process 2 weeks after the previous colony but at a near-identical life-history stage (mean initial workers ± SD = 39 ± 16.8). For 2 weeks prior to release for real-world foraging, a mean of nine (range 7–13) recently emerged workers from the colony underwent cognitive testing in a four-arm radial arm maze (RAM; Figure 1B). Originally developed for rodent toxicology, the RAM is a win-shift paradigm in which all arms are initially baited, and maximum efficiency is achieved by avoiding revisits within a trial (median test trial duration in our paradigm = 3.4 min). Longer-term memories of visited arms are not useful because all arms are rebaited between trials. Previous studies have shown that bees solve the RAM and analogous tasks by remembering and avoiding depleted locations,^36^, ^37^, ^38^ and we additionally provide a validation for a version of our own set-up in Figure S1A. For each subject, we included nine “training” bouts (based on emergence of asymptotic performance in Samuelson et al.^38^) so that bees could learn the win-shift nature of the task, before assaying final performance as the mean total number of errors (revisits to depleted arms) within each of the last three “test” bouts (henceforth RAM score). We obtained RAM scores for 230 bees in total, finding no effect of bee size or age on individual performance (Figure S1B; Data S1A; ΔAIC between null and next best model = 8.77, indicating no impact of these predictors).

After cognitive testing was complete for a given colony, tested workers were tagged with an RFID chip and screened for gut parasites—which may compromise cognition^41^—but no infections were found. Each colony was then given through-the-wall access to the external environment (Figure 1C), comprising broadleaved mixed woodland and parkland surrounded by suburban housing and gardens (Figure 1D). We recorded nectar and pollen foraging efficiency by individually weighing bees on entry and exit for 8 days across the 2 weeks following release (∼6 h per day), sampling ∼47% of each bee’s total foraging career. At the end of this period, external foraging began for the next colony, although we continued to monitor the activity of any surviving tagged workers using RFID data to establish longevity. Of our tested bees, 144 (63%) foraged in the wild for nectar, pollen, or both (nectar trips, n = 1,202 trips by 134 bees; pollen trips, n = 526 trips by 91 bees).

### Foraging efficiency

**Figure 2:**
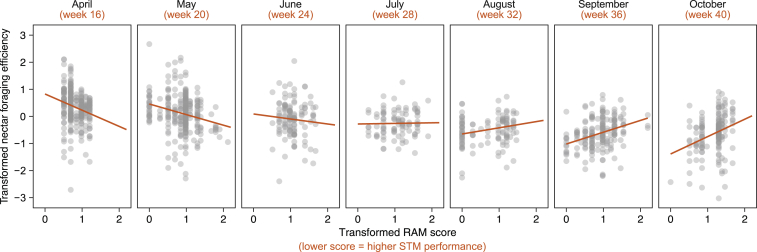
Seasonal reversal in bee STM and foraging efficiency relationship Partial residual plots from a linear mixed-effect model including an interaction between bee RAM score and week of year (n = 1,202 nectar foraging trips by 134 bees). Fitted lines indicate the relationship between RAM score and nectar foraging efficiency at 4-week intervals, holding the effect of other numeric predictors constant at their median (covariates) or mode (factors). Both variables are presented, as analyzed, on transformed scales (ORQ normalization and log(n + 1), respectively); for reference, untransformed nectar values range from −6.75 to 14.85 mg/min and RAM score 0–8 errors.Negative nectar values result from bees leaving with more nectar than they return with.^22^ See also Figure S2 for partial residual plots of additional model covariates, Figure S4 for model validation, and Data S1B for model selection outcomes.

Because we sequentially replaced foraging colonies every 2 weeks with new, standardized, parasite-free colonies that had never previously foraged outside, individual bees in our setup did not themselves experience seasonal change. Thus, we could probe the relationship between RAM score and foraging efficiency continuously across the foraging season, without potentially confounding variables such as bee age, prior experience, colony age, and colony size varying systematically over time. We found that for nectar-collecting trips, the relationship between RAM score and foraging efficiency reversed in direction across the foraging season (Figure 2; Data S1B; linear mixed-effects model [LMER] containing RAM score × week interaction: ΔAIC to next best model = 4.7; interaction estimate ± 95% confidence interval = 0.05 ± 0.02 to 0.09). Bees with better RAM scores collected more nectar/minute in spring but less in summer, compared with bees with poorer RAM scores.

In accordance with previous results,^22^ foraging efficiency also varied within individual lifetimes, whereby bees exhibited an increase in nectar foraging efficiency at the start, and a decrease toward the end, of their foraging careers, presumably as they gained experience and then underwent foraging-driven senescence (Figure S2A; estimate ± 95% CI = −0.03 ± −0.04 to −0.02). Larger bees were also more efficient foragers (Figure S2B; estimate ± 95% CI = 0.34 ± 0.15 to 0.52), and all bees foraged more efficiently on cooler, more humid days (Figure S2C; estimate ± 95% CI = 0.01 ± 0.01 to 0.02). Importantly, none of these factors were colinear with RAM score (see Figure S3 for collinearity plot for all predictors). Neither year of the experiment (Figure S2D; estimate ± 95% CI = −0.28 ± −0.58 to 0.04) nor bee age at initial release (Figure S2E; estimate ± 95% CI = −0.03 ± −0.06 to 0.001) had significant impacts. Pollen foraging efficiency is not expected to vary as drastically with RAM score as nectar foraging because flowers typically contain more pollen than can be extracted in a single visit.^42^ Avoidance of visited flowers within patches is thus less relevant, and accordingly, RAM score did not predict pollen foraging efficiency (Data S1C; generalized LMER [GLMER]: ΔAIC between intercept-only null model and next best alternative = 2.62).

### Survival and lifetime foraging effort

Since our bees were RFID-tagged, we could also relate RAM performance to survival and total lifetime foraging effort (total number of trips), again across the whole season. However, both variables were solely predicted by age, with bees that were older on release dying sooner (Cox proportional hazard model: estimate ± 95% CI = 0.07 ± 0.02 to 0.12) and thus conducting fewer trips (GLMER: estimate ± 95% CI = −0.35 ± −0.55 to −0.15). Adding RAM score to the models did not sufficiently improve fit in either case (Data S1D and S1E). Additionally, comparisons of tested and non-tested control bees revealed that cognition testing itself had no measurable impact on either the foraging performance or survival of bees (Data S1F–S1H).

### Floral seasonality

**Figure 3:**
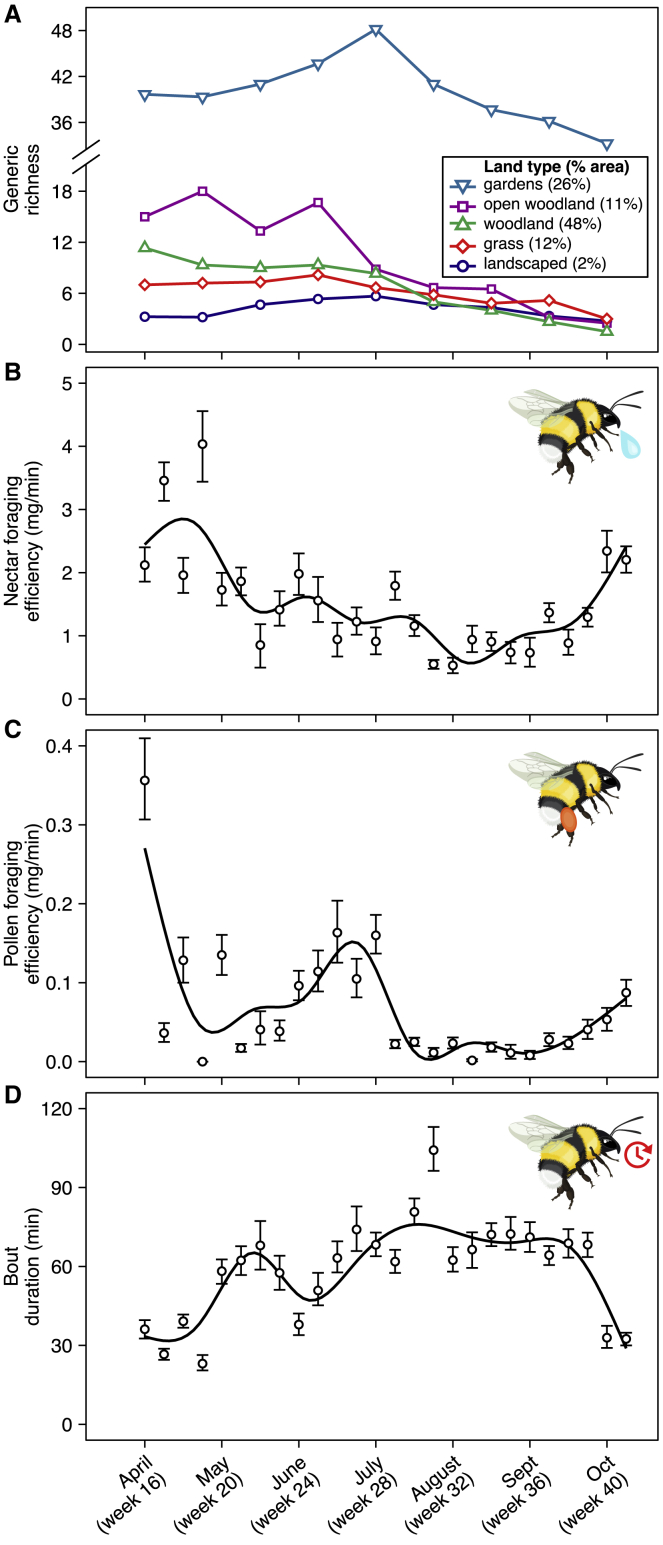
Seasonal variation in floral resources and foraging (A) Shifts in floral generic richness across the foraging season. Each point represents a 3-week average (or 2 weeks for the final survey) based on both years of the study (except surveys 1–4 for gardens, open woodland, and woodland; STAR Methods). Percentages in legend indicate proportion of each land type within the survey area. (B–D) Nectar foraging efficiency, pollen foraging efficiency, and bout duration for whole colonies (n = 6,616 foraging trips) across both years of the foraging season (weekly mean ± 95% CI; trend lines fitted using a generalized additive model [GAM] smoother function). See also Figure S4 for generic richness of bee-collected pollen and Data S1I and S1J for model selection outcomes and parameter estimates.

Our results suggest that bees performing better on the RAM are more efficient nectar foragers in spring but not in summer, indicating that the benefits of short-term memory (STM) are realized in rich rather than in sparse environments. However, this claim rests on the assumption that floral resources were more abundant in spring within our study. To confirm this expected seasonal pattern in floral phenology, we combined land classification techniques with transect and quadrat sampling to measure floral generic richness within a 500 m radius around our colonies, spanning mixed broadleaved woodland and parkland, local public green spaces, and residential gardens. Weekly surveys revealed an overall decline in the number of genera in flower toward the end of the season for all land types (Figure 3A; all statistics given in Data S1I and S1J). Floral resources in wooded areas, which is the dominant land type in our survey area (woodland = 48%, open woodland = 11%), showed the sharpest linear decline from a peak in spring; others, such as private gardens (26%), increased toward a peak in early summer before declining by the end of summer. This pattern is also mirrored in the gross foraging trends found for all bees across the season: nectar and pollen foraging efficiency peaked in spring and was lowest at the end of summer, albeit with a brief revival in early autumn (Figures 3B and 3C). Bout duration (Figure 3D), which correlates with resource availability in bumblebees,^43^ followed the same pattern. Finally, identification of pollen loads collected by bees in the second year of our experiment showed a gradual decline in floral generic richness from a spring peak of fifteen genera to a late summer trough of one genus (Figure S4). Together, these data indicate that spring foraging environments were plentiful in comparison with the relatively depauperate summer.

## Discussion

**Figure 4:**
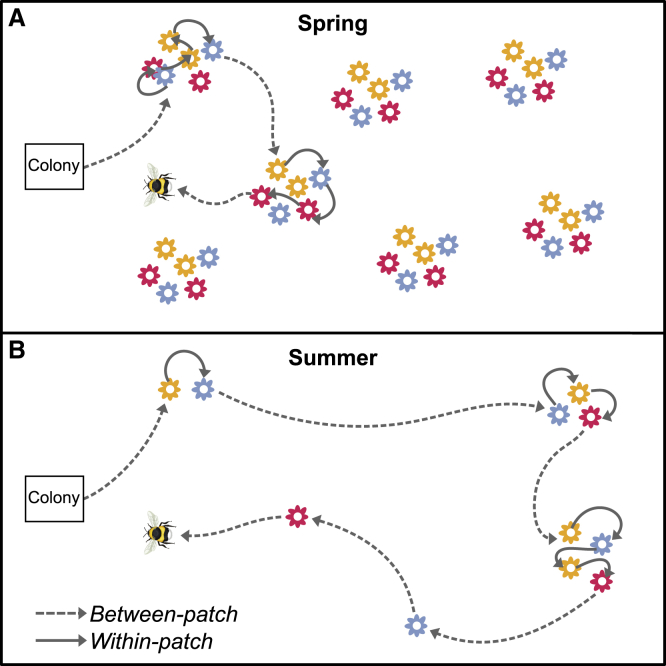
Hypothesized bumblebee foraging patterns across season (A) Spring foraging is potentially characterized by short flights between rich patches close to the nest, and bees make many successive flights to flowers within single patches. (B) In contrast, summer foraging is potentially dominated by long flights between patches that are further from the nest, with possibly fewer flights to flowers within single patches.

Our findings show that for foraging bumblebees, within-patch memory performance as assayed through a RAM predicted foraging efficiency in a rich spring environment, but not in harsher summer conditions. This finding contrasts with studies in other species that have linked cognitive abilities to fitness proxies in conditions of food scarcity (e.g., high-elevation winters^9^^,^^10^), but it is consistent with hypotheses that relate phases of memory to foraging efficiency in bees.^28^^,^^35^^,^^39^^,^^44^ For social bees, foraging is characterized by short flights from flower to flower within a patch (such as a flowering tree or shrub), interspersed by longer between-patch trips when rewards diminish (Figure 4). The RAM seeks to mirror the former, within-patch context, where the ability to briefly hold a memory of the most recently visited flower may both allow bees to avoid revisits, to decide whether to stay in a patch and/or to match the flower’s image to the next one encountered before the memory of it degrades,^28^^,^^35^^,^^45^^,^^46^ and so increase within-patch foraging efficiency. In contrast, between-patch efficiency is more likely to depend upon long-term memories of rewarding locations (which bees may return to over the course of several days^47^) and flower species.^28^ We hypothesize that in spring, when resource patches are rich and plentiful near to the colony, within-patch efficiency savings may be more detectable than in summer, when such effects are potentially dwarfed by long travel times to and from patches and less time is spent within them (Figure 4). In other words, under plentiful conditions, overall foraging efficiency may be predominantly determined by within-patch efficiency, and in sparse environments, between-patch efficiency might be more important. Accordingly, previous work in the same species^19^ has shown that performance in a task requiring longer-term memory predicted foraging efficiency at the height of summer (but see Evans et al.^32^).

An increase in the relative importance of within-patch efficiency savings with patch density could explain why high-scoring bees performed relatively well in spring, but not why strong RAM performance negatively predicted foraging efficiency (rather than simply being inconsequential) in summer. Why should the ability to remember flowers over short timescales come at a cost to foraging efficiency when resources are sparse? One possibility is that investment in STM comes at a cost to other cognitive traits. In *Drosophila*, the short-term ability to form single-trial memories (or multiple trials with very short inter-trial intervals—termed anesthesia-resistant memory [ARM])—trades off against the long-term memory (LTM), such that flies with good ARM have poorer LTM.^48^ However, this trade-off has not yet been explored in bees. Alternatively, STM investment may trade-off against physiological or metabolic traits, given that investment into cognition has been found to come at significant constitutive and induced costs.^12^^,^^49^ We found no significant effect of STM on individual survival, but we cannot rule out the possibility of sublethal costs of cognitive investment that emerge in summer. For example, when flight times between patches are relatively long (Figure 4B), high-scoring bees may pay costs manifested through flight efficiency or the need to consume more of the nectar that is collected while foraging. Future research could productively investigate the link between such metabolic costs and investment in memory because sublethal stressors can critically compromise colony reproductive success.^50^

Although variation in the benefits afforded by cognitive traits have been hypothesized to vary with environmental conditions^1^, ^2^, ^3^, ^4^—even within individual lifetimes—direct demonstrations of such fluctuations in potential selective value have not previously been performed. Here, we have provided evidence that the benefit of a cognitive trait can indeed vary considerably, even within a relatively short single foraging season, becoming apparent in plentiful, rich floral environments but reversing in depauperate ones. For bumblebees, the timescale of this reversal did not fall within the expected lifespan of individual workers, who typically live for only a few weeks in the wild^22^ but instead within the lifespan of the colony. Thus, future studies could evaluate the possibility that worker cognitive abilities may even vary with the stage in the foraging season at which an individual emerges, allowing bees to capitalize upon spring-flowering trees and hedgerows that are known to be key to colony survival and performance.^51^ Assaying the repeatability of RAM scores would also provide a step toward evaluating the potential that selection acts on memory differentially according to food availability. In longer-lived animals that will experience variability within a single lifetime, such patterns might be expected to compromise selection for certain cognitive traits if the costs of maintenance in some periods outweigh the benefits in others.^52^

Although previous work within the ecological intelligence framework has placed emphasis on the potential role of food scarcity in driving cognitive evolution,^5^^,^^9^, ^10^ our study suggests that plentiful environments, where food is easy to find, could be just as important a selective environment for certain cognitive traits. In our study, individuals that performed well at a within-patch memory task excelled at foraging in such rich environments, when all individuals fared relatively well. However, such effects will be specific to the cognitive trait in question,^28^ and longer-term memory formation may well show the opposite pattern, both in our *Bombus* system and more generally. For example, in populations of black-capped chickadees (*Poecile gambeli*) that live at higher elevation and thus experience harsher winters, performance on a task that requires longer-term memory than the one-trial task described here correlates with overwinter survival.^10^ Likewise, a previous study in bumblebees that focused on summer conditions found that longer-term memory correlated with colony-level foraging efficiency.^19^ In both these cases, food is likely to be sparse, and longer-term memories of cache/forage locations may well be more important than the shorter-term memories that could improve within-patch efficiency. Overall, we have found strong support for the main tenet of the ecological intelligence framework: that the benefits of cognitive investment vary with the foraging environment. Our study thus points to the major role that ecological differences possibly play in generating and maintaining the considerable intraspecific cognitive variation observed across the animal kingdom.^6^^,^^7^^,^^21^

## STAR★Methods

### Key resources table

**Table:** 

REAGENT or RESOURCE	SOURCE	IDENTIFIER
**Deposited data**		

All supporting data arising from study	This paper	https://doi.org/10.6084/m9.figshare.19103177.v2
Weather station data	Imperial College London, Silwood Park	https://www.imperial.ac.uk/silwood-park/research/field-experiments/silwood-weather

**Experimental models: Organisms/strains**		

Buff-tailed bumblebee, *Bombus terrestris*	BioBest (via Agralan)	BB121040-CF2

**Software and algorithms**		

QGIS	QGIS Development Team	https://www.qgis.org/
BORIS	BORIS Team	https://www.boris.unito.it
R	The R Foundation	http://www.r-project.org/
Original code used for statistical analysis in R	This paper	https://doi.org/10.6084/m9.figshare.19103177.v2

**Other**		

Radial arm maze	This paper	N/A
RFID system	MicroSensys GmBH	Bee Identification System: iID2000
mic3-TAG 16k RFID chips	MicroSensys GmBH	iID-2000-G
Advanced portable Balance Scout STX 120g/1mg	Ohaus	STX123

### Resource availability

#### Lead contact

Further information and requests for resources and reagents should be directed to and will be fulfilled by the lead contact, Christopher Pull (christopher.pull@biology.ox.ac.uk).

#### Materials availability

This study did not generate new unique materials.

### Experimental model and subject details

We used commercial colonies of UK-native *Bombus terrestris audax* (BioBest, Belgium, distributed by Agralan Ltd, UK), housed in two-chamber plastic nestboxes (28 [l] x 16 [w] x10.5 [h] cm). Commercially supplied colonies are reared from domestic *Bombus* lines and queens had emerged as gynes, hibernated, and founded colonies in identical controlled indoor conditions. All colonies were young on arrival (mean initial number of workers ± SD = 39 ± 16.8), and only worker bees that emerged post-arrival (identified through tagging all bees with numbered discs on arrival and new bees after emergence [Abelo, UK]) were tested in our cognitive assay. Prior to cognitive testing, colonies were fed an *ad libitum* supply of inverted sugar syrup via an in-nest feeder (45% [w/v]; Thorne, Windsor, U.K.), with one 1.5g pollen ball (2:1 honeybee-collected pollen: 45% inverted sugar) added daily (two on Fridays; none on weekends). During cognitive testing, pollen feeding continued, but syrup feeders were removed and between 10-15 ml (depending on colony size and stores) was pipetted into nectar pots per weekday evening. Colonies were not fed once given outside access.

### Method details

#### Experimental overview

From April to October in 2018 and 2019, following a staggered design (Figure 1A), we performed laboratory-based cognitive testing on 230 worker bees from 25 commercially sourced colonies (Figure 1B; *n* = 12 in 2018 and 13 in 2019; one colony removed in April 2018 due to colony death pre-release). After a two-week testing period, tested bees were screened for gut parasites and RFID-tagged, and colonies were connected to a through-the-wall external access hatch (Figure 1C). Nectar and pollen foraging efficiency was monitored for two weeks (approx. 6h per day, 4-5 days per week). At the same time, testing began for the next colony in the cycle (Figure 1A). After foraging efficiency recording had ceased, colonies were permitted to continue foraging for another two weeks, during which time survival and foraging activity (but not foraging efficiency) was recorded through automated RFID readers. Throughout, we conducted weekly surveys of the land surrounding the foraging colonies, to record changes in the abundance and diversity of floral resources over the seasons (Figure 1D).

#### Radial arm maze

We used a radial arm maze (Figure 1B) to assess the STM of individual bees. The RAM is a win-shift paradigm in which all arms are initially baited with food rewards that are not replaced upon removal, within one bout. Revisits to depleted arms constitute “errors” and the number of errors within a single foraging bout is the measure of performance.^53^ The RAM is therefore an ecologically relevant task that mimics natural nectar foraging within a flower patch. The task places demands on within-bout memories because avoiding revisits within bouts can increase performance (median bout duration in our set-up = 3.4 mins) but remembering visited flowers between bouts – when all arms are rebaited – cannot (see below for empirical validation of the RAM). Our RAM consisted of an octagonal four-arm array in which differentiation of the arms was possible through a laminated black and white panoramic image of the laboratory at the ends of each arm. Rewards were accessed via removable blue, rectangular Perspex platforms – henceforth “flowers” – at the end of each arm (colour number = 744; 7.5 [l] x 3 [w] x 0.5 [h] cm) that slotted through holes in the maze wall (Figure 1B). Bees retrieved a sucrose reward (see “cognitive testing”) by alighting on flowers and inserting their proboscises through small holes in the RAM wall (Figure 1B inset). After each landing, the flower was removed and replaced with an identical clean alternative to rule out the use of scent marks.

#### Cognitive testing

Cognitive testing began 12 days after arrival in the laboratory (to allow bees of known age to emerge) and commenced with a group training period (∼1h) whereby bees were allowed to forage freely in the RAM and all arms were continually rewarding (an *ad libitum* supply of 2M sucrose solution), and to enter and leave the arena at will. Motivated foragers were then tested in the RAM alone for 12 foraging bouts, during which all arms were baited with 20 μl of 2M sucrose solution that was not replaced within a bout (except the last non-depleted arm, when bees were fed to repletion). After each landing, platforms were replaced with identical clean, unrewarded replacements. Access to the nest during testing was prevented via sliding shutters, unless a bee spent >30 s trying to return or attempted to return more than twice within a bout (to maintain foraging motivation). Consequently, in some bouts not all arms were visited. All bouts were filmed for later video analysis using BORIS video analysis software.^54^ One researcher watched and coded all videos but was blind to the foraging performance of the bees; moreover, RAM score was only calculated once all video coding was completed, preventing any unconscious bias.

#### Parasite screening and RFID chips

We screened faecal samples from all tested bees for the presence of gut parasites (*Apicystis bombi*, *Nosema bombi* and *Crithidia bombi*) at the end of the two weeks of cognition testing (Nikon e50i) and measured intra-tegula distance as a proxy for overall size, as in other studies (e.g., Raine and Chittka^19^ and Samuelson et al.^38^). An RFID chip (Microsensys GmbH) encoding a unique 16-digit identification number was also superglued (Loctite) to the thorax. We performed the same procedure for a cohort of non-tested bees from each colony that were observed foraging during group training (mean of four bees per colony, total *n* = 99), to confirm that our testing regime had no effect on subsequent foraging efficiency.

#### Measuring foraging performance

We measured the foraging success of individual bees on exiting and re-entering the colony using weight-averaging scales for moving subjects (mean of three repeat measurements with 2s averaging each and accuracy of ± 2 mg; Advanced portable balance Scout STX123 120g; OHAUS Corporation) and their lifetime foraging activity and survival using an RFID system (MicroSensys GmBH, Figure 1C). Per trip nectar values were calculated by subtracting the bee’s outbound weight from her inbound weight, minus the weight of any pollen. We removed pollen from one leg of the bee – via a trap door in the tube – and weighed and froze (-20 °C) the pollen for later identification (see below). The weight of the pollen was doubled and subtracted from the bees’ weight. On entry and exit, bees passed through two RFID readers (Microsensys GmbH) that recorded identity and travel direction. We collected foraging performance data for ∼ 6 hours per day, four-five days a week, for two weeks per colony. In total, 6616 were trips recorded across all colonies and bees. For analysis, nectar trips (*n* = 1202 trips by 134 bees) were defined as those where < 3 mg of pollen was collected, and pollen trips > 3 mg (*n* = 526 trips by 91 bees), based on histograms of foraging data. Any trips that were less than 7 min in duration were not included in the analysis, because such trips are more likely to represent orientation flights or waste disposal.

Colonies then foraged for a further two weeks with only RFID data collection to assay survival, in which time >99% of RFID-chipped bees eventually failed to return to the colony and were presumed dead. We affixed brightly coloured plastic cones onto the outdoor entrance of nests and replaced pollen that we removed daily for microscopic pollen identification using a local reference collection with equal quantities of honeybee collected pollen (Koppert, UK). Colonies were euthanized at the end of four weeks of foraging.

#### Pollen identification

Pollen loads were defrosted, vortexed for 60 s and suspended in water (1 mg pollen:10 μl pure water); 1 μl was added to a glass slide and heated at 50 °C for 20 s, followed by two drops of melted glycerine with fuschine dye (Brunel Microscopes). The slide was covered with a slip and left to cure at 50 ºC for 30 seconds. We discounted any floral morphotype where the grain count was < 50 grains per section of slide that was counted. Morphotypes were microscopically identified to genus level using a combination of sources^55^^,^^56^ and the pollen reference collection at Royal Holloway University of London.

#### Floral resource surveys and weather data

We used QGIS to (*i*) classify the 500 m radius surrounding our colonies into broad land use types likely to contain forage: woodland, grassy woodland, grassland and landscaped and (*ii*) select sampling sites within these categories. Additionally, we arranged access to private suburban gardens (total sites per land use type = 12, except grass = 24 and gardens = 11). Surveys were performed for one day each week, whereby each site was visited once every three weeks on a rotational basis. Methods were customized to each land-use type: in grass and landscaped areas, we sampled 0.25m^2^ quadrats; in woodland and open woodland we surveyed 30 m transects; in gardens we counted every genus of plant in flower. In year one, we utilized a different approach to record transect and garden data that was discarded after the 4^th^ survey; hence we only include transect and garden data from year two in survey blocks 1-4 of Figure 3A. A three-week average was calculated using generic richness data from all three sets of sites for the same survey period in both years for graphical representation in Figure 3A. Local meteorological data was collected from a weather station located ∼ 8 km away at Imperial College London, Silwood Park. Hourly weather data (temperature, humidity, and wind speed) was averaged to produce a daily mean for analysis.

#### RAM validation

In an additional experiment, we tested whether bees perform better on the RAM than using stereotypical movement rules or by chance alone. Using the same procedure as before, we tested 20 bees from four colonies on an eight-arm version of our RAM (to increase the difficulty of the task). Each bee performed ten training bouts, to learn how to use the maze, followed by ten test bouts once performance had plateaued. Following Brown and Demas^36^ and Samuelson et al.,^38^ we used data from the test bouts to calculate the general probability of moving from each flower to each of the other flowers (e.g. from F1 to F2, from F1 to F3 etc.), creating an individual transition matrix for each bee. We used these transition matrices to create a simulant dataset of 20 bees, whereby the movements of each simulant were derived from one of the 20 transition matrices. This process was repeated 10,000 times to create 10,000 simulant datasets. For each simulant dataset, and for the observed performance of the 20 real bees, we fitted a generalised linear mixed model (GLMM) in which body size and bee age were fixed factors, and individual bee as a random intercept effect, extracting the intercept in each case. Calculated p-values based on the percentile of the simulated distribution in which the intercept for the observed data fell (Figure S1A).

A similar process, using randomly generated transition matrices, was followed to estimate likely performance following random selection of platforms.

### Quantification and statistical analysis

All statistical analyses were conducted using R version 4.1.0 and an information-theoretic approach; data and code are available.^57^ For foraging and survival models, we built a candidate model that contained every hypothesized covariate (see below and Data S1 for model descriptions) and compared this to (*i*) the same model, but with RAM score as a predictor, (*ii*) the same model but including an interaction between RAM score and week (*iii*) a null model containing only the intercept and random factors. Model selection was based on ΔAIC or ΔAICc (depending on sample size), where a cut-off of >2 was used to identify the best model. If nested models had comparable fit the simplest model was selected.

We analysed whether the RAM score of bees was affected by bee age, size, or participation in group training (*n* = 230) using a linear mixed effects regression (LMER). We included the RAM score (average number of errors on test abouts) as the response and bee size, the age of the bee when tested, and whether the bee had engaged in group training on the morning of testing as covariables. We log(n+1) transformed RAM score due to non-normality of model residuals. See also Data S1A for RAM score model selection outcomes.

We modelled nectar foraging efficiency using a LMER that included foraging efficiency as the response (mg/min; ordered quantile normalization transformation using the BestNormalize package^58^ due to heteroscedasticity) and week-of-year, RAM score (log(n+1) transformed to reduce influence of outliers) and their interaction as our predictors of interest, with initial bee age (at time of release), bee size, year of experiment, foraging experience (number of days since foraging began), and a composite “weather” score as covariates. The composite weather score was produced via principal component analysis to reduce temperature, humidity, and wind speed into a single component (∼ 84% variation captured). To account for collinearity between weather scores and week, weather scores in the main analysis are the residuals of generalized additive model (GAM) predicting weather based on week; thus, any reported effects of “weather” reflect those that occurred in addition to effects of week. We included foraging experience as a quadratic polynomial to account for its non-linear effect, which improved model fit. For each bee, we fit random intercepts that interacted with random slopes for experience (because improvement in performance could vary between bees and is likely affected by starting performance) and a random intercept per colony. To model pollen foraging efficiency, we used a GLMER with log-link Gamma errors; foraging experience as a linear predictor and uncorrelated random slopes and intercepts fit this data best, otherwise the model was identical to the nectar LMER. See also Data S1B and S1C for nectar and for pollen foraging model selection outcomes.

To analyse survival data, we used a Cox proportional hazards model with week of year, RAM score and their interaction as our predictors of interest, and bee age (at time of release), bee size, composite weather score, and year of experiment as covariates, including colony as a random effect using the shared frailty function. We used a GLMER with Poisson error distribution to model lifetime foraging effort, including number of RFID-recorded trips as the response and an observation-level random effect to account for overdispersion^59^; all other covariates were identical to the nectar foraging model and colony was included as a random intercept. See also Data S1D and S1E for survival and lifetime foraging effort model selection outcomes.

We built several models to investigate if our cognition testing regime had a subsequent impact on the foraging performance and survival of tested bees. Firstly, we fit a LMER with Gaussian error distribution that had nectar foraging efficiency (mg/min) per foraging trip as the response, with treatment (control or cognition tested) as a main effect, and bee size, initial age, year of experiment, composite weather score, days since release as a quadratic polynomial and week of year as covariates. We transformed the response using QRQ normalization due to heterogeneity. We fitted random intercepts per bee that interacted with random slopes for experience and a random intercept per colony. Secondly, we ran a GLMER with Gamma error distribution and log link that had pollen foraging efficiency (mg/min) per foraging trip as the response, and the same covariates as above, except day since release was included as a linear term and the interaction between the random intercepts and slopes was removed. Lastly, we fit a cox proportional hazards model that included treatment as a main effect and bee size, initial age, composite weather score, year of experiment and week as covariates, with colony as a random effect using the frailty function. See also Data S1F–S1H for control vs. treated bee nectar, pollen, and survival model selection outcomes.

To analyse generic floral richness, we built linear regressions that included weekly generic richness as the response, season as a linear predictor or quadratic polynomial (depending on fit), and year of experiment as a covariate. For gardens and woodland data, we applied a log(n) and ORQ transformation respectively to the response to achieve normality in model residuals. See also Data S1I and S1J for generic richness model selection outcomes and parameter estimates.

For mixed effects modelling, we used the ‘lme4’ package^60^ and checked all model assumptions by viewing plots of both raw data and of the distribution of model residuals, testing for unequal variances, testing for the presence of multicollinearity, testing for over-dispersion, and assessing models for instability and influential observations, utilising functions in ‘influence.ME’ and ‘performance’ packages.^61^^,^^62^ Importantly, we followed standard data exploration protocols to identify potential collinearity in our predictors, including individual bee size, age and RAM score, both pre- and post-analysis,^63^ through (*a*) visual inspection of data and model residuals and (*b*) estimation of variance inflation factor, revealing no issues (all VIFs < threshold of 3^63^) except for the weather variables (see above). For the survival model, we plotted residuals, tested for non-proportional hazards, and assessed the model for influential observations.

## Data Availability

• All supporting data have been deposited at FigShare and are publicly available as of the date of publication. DOIs are listed in the key resources table. • All original code has been deposited at FigShare and is publicly available as of the date of publication. DOIs are listed in the key resources table. • Any additional information required to reanalyse the data reported in this paper is available from the lead contact upon request.

## Supplemental information

Document S1. Figures S1–S4

Data S1. Tables of candidate model sets, model selection outcomes, and model parameter estimates, related to STAR Methods and Figures 1–3, S1, and S2 Where relevant: an ∆AIC > 2 was used to select the final model (highlighted in bold); if more than one model was within 2 ∆AIC points, then the simplest (least terms) was chosen. All models included the same random effect structure and, except the intercept-only null model, the same set of covariates. A) Nested candidate model set and model selection outcome for a LMER investigating the relationship between RAM score and bee age, size, and participation in group training. B) Nested candidate model set and model selection outcome for an LMER investigating the relationship between season (week of year) and RAM score on nectar foraging efficiency. C) Nested candidate model set and model selection outcome for a GLMER investigating the relationship between season (week of year) and RAM score on pollen foraging efficiency. D) Nested candidate model set and model selection outcome for a Cox proportional hazards model investigating the relationship between season (week of year) and RAM score on bee survival. E) Nested candidate model set and model selection outcome for a GLMER investigating the relationship between season (week of year) and RAM score on lifetime foraging effort (total number of bouts recorded by RFID system). F) Nested candidate model set and model selection outcome for a LMER investigating the effect of cognition testing on the nectar foraging efficiency of RAM tested and control bees. G) Nested candidate model set and model selection outcome for a LMER investigating the effect of cognition testing on the pollen foraging efficiency of RAM tested and control bees. H) Nested candidate model set and model selection outcome for a Cox proportional hazards model investigating the effect of cognition testing on the survival of RAM tested and control bees. I) Nested candidate model set and model selection outcome for linear regressions investigating the effect of season (week of year) and year of experiment on floral generic richness across survey land types. J) Parameter estimates ± 95% CIs for the best fitting model per land type.

Document S2. Article plus supplemental information

Video abstract
