# Supplementary material for: Ecology dictates the value of memory for foraging bees
Source: Curr Biol. 2022 Oct 10;32(19):4279–4285.e4. doi: 10.1016/j.cub.2022.07.062 (PMC9616731; doi:10.1016/j.cub.2022.07.062)
Supplement: Document S1. Figures S1–S4 [file mmc1.pdf]

Current Biology, Volume *32*

Supplemental Information

Ecology dictates the value

of memory for foraging bees

Christopher D. Pull, Irina Petkova, Cecylia Watrobska, Grégoire Pasquier, Marta Perez Fernandez, and Ellouise Leadbeater

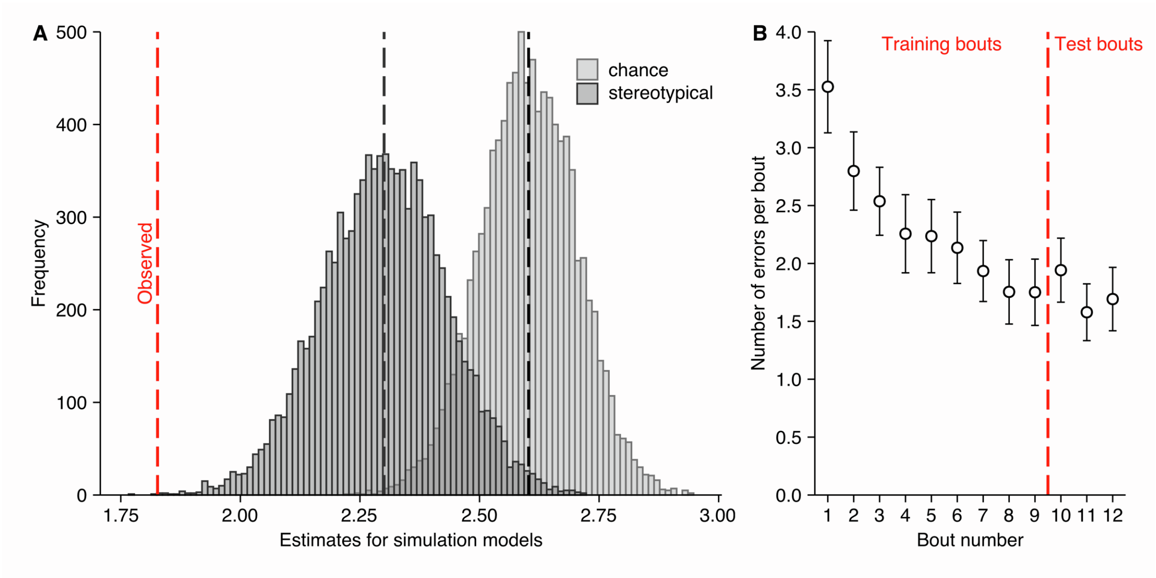

Figure S1. RAM validation and performance, related to Figure 1. (A) Bees perform better than chance alone or by using stereotypical movement rules on an eight-arm radial arm maze. For simulated estimates, vertical lines represent mean values whilst the red vertical line represents the intercept of a model based on observed data (see methods); frequency bars represent the intercepts from the same model obtained from the 10000 simulated datasets (*n* = 20 bees). **(B)** Bee performance on a four-arm RAM. Number of errors during the last three test bouts (10-12) were averaged to produce a mean RAM score for subsequent analysis. Dots ± error bars show mean number of errors per bout ± 95% CIs (n = 230 bees). RAM score was unaffected by bee age, size, or participation in group training (Supplementary Table 1; ΔAIC between null and next best model = 8.77).

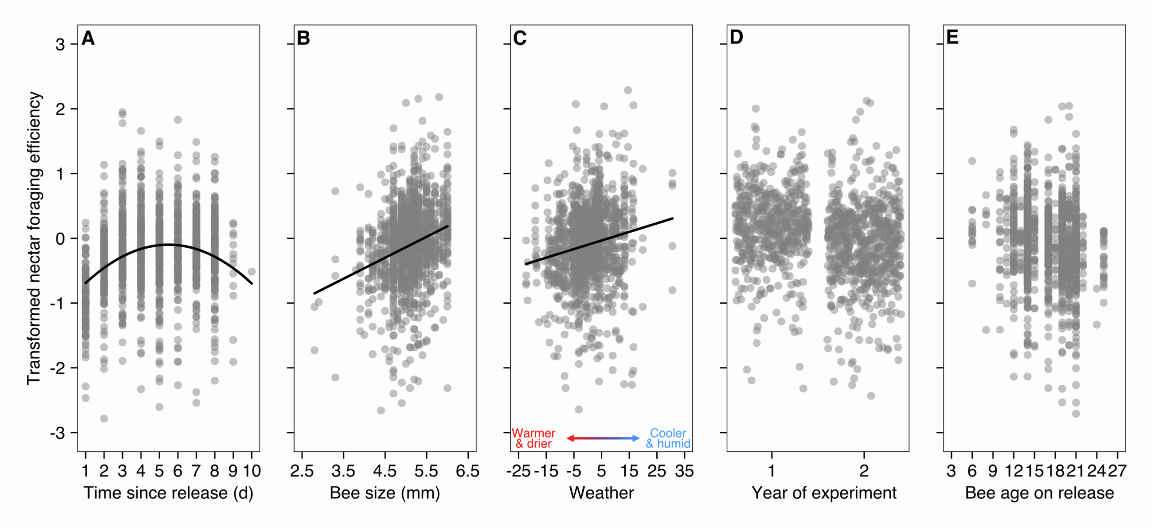

Figure S2. Influence of covariates on nectar foraging efficiency, related to Figure 2. Partial residual plots for covariates from a linear mixed effect model with an interaction between bee RAM score and week of year (n = 1209 nectar foraging trips; RAM score x week interaction displayed in Fig 2). Fitted lines (a-c) included where there is significant relationship between the covariate and nectar foraging efficiency, while holding the effect of other numeric predictors constant at their median and by setting year to “two” (most common value). Nectar foraging efficiency is presented, as analyzed, on transformed scales (ORQ normalization); for reference, untransformed nectar values range from -6.75–14.85 mg/min.

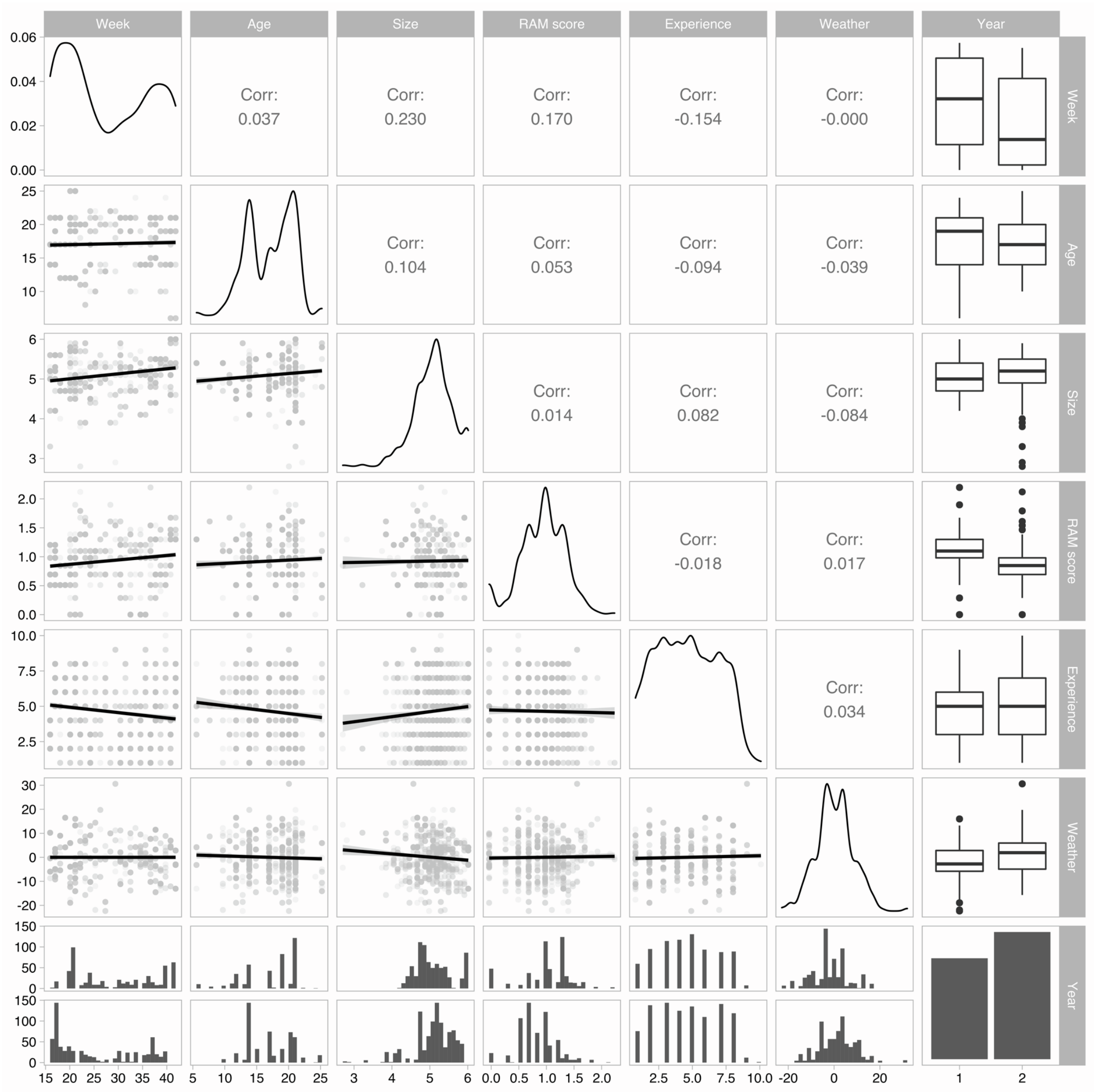

Figure S3. Correlogram of covariates used in final foraging efficiency models to assess for potential multicollinearity, related to Figure 2. Density plots show distribution of focal covariate, scatterplots all pairwise correlations between continuous covariables, histograms and boxplots the distribution of continuous covariates across the two years, and Corr numbers are Pearson's correlation coefficient values. No correlations are present and post-model assessment revealed that all variance inflation factors (a measure of multicollinearity) are well below the threshold of 3.

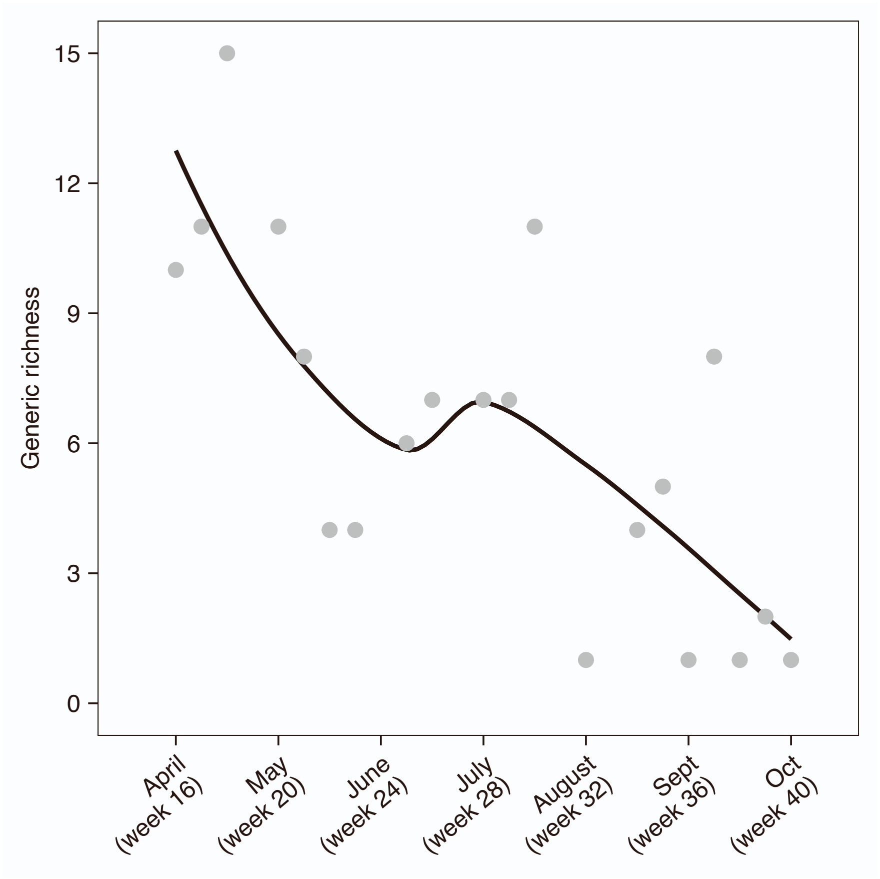

Figure S4. Generic richness of pollen samples collected from cognition-tested bees in second year of experiment, related to Figure 3. Dots represent summed generic richness values for the week pollen samples were collected in, and smoothed trend line was fitted via LOESS.
